# Regulation of V-ATPase by Jasmonic Acid: Possible Role of Persulfidation

**DOI:** 10.3390/ijms241813896

**Published:** 2023-09-09

**Authors:** Magdalena Zboińska, Luis C. Romero, Cecilia Gotor, Katarzyna Kabała

**Affiliations:** 1 Department of Plant Molecular Physiology, Faculty of Biological Sciences, University of Wrocław, Kanonia 6/8, 50-328 Wrocław, Poland; magdalena.zboinska@uwr.edu.pl; 2 Instituto de Bioquímica Vegetal y Fotosíntesis, Consejo Superior de Investigaciones Científicas, Universidad de Sevilla, C. Américo Vespucio, 49, 41092 Sevilla, Spain; lromero@ibvf.csic.es (L.C.R.); gotor@ibvf.csic.es (C.G.)

**Keywords:** cadmium, H_2_O_2_, H_2_S, jasmonic acid, persulfidation, proton pump, vacuolar H^+^-translocating ATPase

## Abstract

Vacuolar H^+^-translocating ATPase (V-ATPase) is a proton pump crucial for plant growth and survival. For this reason, its activity is tightly regulated, and various factors, such as signaling molecules and phytohormones, may be involved in this process. The aim of this study was to explain the role of jasmonic acid (JA) in the signaling pathways responsible for the regulation of V-ATPase in cucumber roots and its relationship with other regulators of this pump, i.e., H_2_S and H_2_O_2_. We analyzed several aspects of the JA action on the enzyme, including transcriptional regulation, modulation of protein levels, and persulfidation of selected V-ATPase subunits as an oxidative posttranslational modification induced by H_2_S. Our results indicated that JA functions as a repressor of V-ATPase, and its action is related to a decrease in the protein amount of the A and B subunits, the induction of oxidative stress, and the downregulation of the E subunit persulfidation. We suggest that both H_2_S and H_2_O_2_ may be downstream components of JA-dependent negative proton pump regulation. The comparison of signaling pathways induced by two negative regulators of the pump, JA and cadmium, revealed that multiple pathways are involved in the V-ATPase downregulation in cucumber roots.

## 1. Introduction

Vacuolar H^+^-ATPase (V-ATPase) is one of the three proton pumps functioning in plant cells. The enzyme is located in the tonoplast and the *trans*-Golgi network/early endosome (TGN/EE), as well as the endoplasmic reticulum, where the complex is assembled [1,2]. Moreover, V-ATPase is found in the endomembrane system of all eukaryotic cells, including animals. It is also evolutionarily the oldest and most complex proton pump [2,3].

V-ATPase is a large, multimeric enzyme composed of thirteen various subunits, named VHA, organized into two sectors: cytoplasmic V_1_ composed of eight different subunits (A–H) and transmembrane V_0_ consisting of subunits a, c, c″, d, and e. In V_1_, several parts can be distinguished: the cylinder-shaped A_3_B_3_ hexamer with three ATP binding sites located at A/B interfaces, the central stalk (DF connected with d), three peripheral stalks (3 × EG), and the stator (C, H, and a). V_0_ contains two important components the proteolipid c-ring and the subunit a with two hemichannels responsible for proton translocation [2].

V-ATPase activity is necessary for the maintenance of fundamental cellular processes such as secondary active membrane transport, endocytosis and vesicle trafficking, protein glycosylation, and autophagy [2]. The knockdown of the main genes encoding V-ATPase is lethal for both animals [4] and plants [5]. In plant cells, the pump mediates many functions performed by the central vacuole, including the regulation of cellular pH and ion homeostasis, as well as the generation of turgor pressure essential for cell expansion [6] and stomatal opening [7].

To maintain optimal metabolic conditions in a constantly changing environment, the activity of vacuolar H^+^-ATPase, which is crucial for cell survival but consumes a large pool of ATP, must be tightly regulated [8]. One of the earliest identified; however, still not clearly elucidated, mechanisms controlling V-ATPase activity is redox regulation. V-ATPase sensitivity to redox potential related to intrasubunit disulfide bond formation between conserved cysteine residues at the VHA-A, near the catalytic center of the enzyme, has been proposed for animals [9], yeast [10], and plants [11]. Moreover, in plants, the S‒S bridge within VHA-E appears as a consequence of the conformational changes induced by ATP binding to the enzyme [12]. Recent proteomic studies revealed that V-ATPase subunits are subjected to other oxidative posttranslational modifications (Ox-PTM) [13]. NO-dependent S-nitrosylation (R-SNO) [14,15], ROS-generated sulfenylation (R-SOH) [16], and H_2_S-induced persulfidation (R-SSH) [17,18] of *Arabidopsis* VHA-A have been confirmed. All these modifications occur on the sulfhydryl (thiol) group of cysteine and are reversible [19,20]. Although the physiological role of these V-ATPase modifications has not been clarified, in general, oxPTMs, similar to other PTMs, can precisely change the conformation and activity of proteins and affect their subcellular localization [20]. In addition, because the same Cys residue can undergo several types of oxPTMs, persulfidation may modulate the S-nitrosylation level [21] or counteract the irreversible oxidation of thiol to sulfinic acid (R-SO_2_H) or sulfonic acid (R-SO_3_H), which in most cases negatively influences protein functioning [22].

Jasmonates, which include jasmonic acid (JA) and its derivatives and conjugates, are involved in both growth and defense processes [23]. JA was found to diminish plant growth. It prevents DNA replication and mitosis [24], represses primary root growth, hypocotyl elongation, and expansion of leaves, cotyledons, and petals; however, on the contrary, promotes trichome formation [25]. On the other hand, it was shown that jasmonate application protects plants against biotic and abiotic stresses, including insect invasion, salinity, UV-B radiation, drought, cold, heat, and heavy metals [26,27]. The mechanism of jasmonate action is related to, among others, increasing the activities of antioxidant enzymes and glutathione content, promoting the accumulation of phenolic compounds and osmoprotectants, such as soluble sugars or proline, modulating stomatal conductance, and regulating the uptake and distribution of macro- and micronutrients as well as toxic ions, such as Na^+^ or Cd^2+^ [25,27]. It has been confirmed that in the signal transduction pathways, JA interacts with other stress hormones (ABA, ethylene, and salicylic acid), kinases, polyamines, and small signaling molecules, such as H_2_O_2_, NO, or H_2_S [23,28,29,30].

To expand our previously proposed model of proton pump regulation in *Cucumis sativus* roots [31], in this study we aimed to explain the role of JA in this process. We showed that JA, similarly to Cd, is involved in the downregulation of vacuolar H^+^-ATPase in cucumber. To determine the mechanism of JA action, the gene expression and protein level of selected VHA subunits were analyzed in plants exposed to this hormone. To verify the possibility that H_2_S and H_2_O_2_ function as downstream components of JA-dependent V-ATPase regulation, H_2_S and H_2_O_2_ levels were analyzed in JA-treated plants. Moreover, V-ATPase activity was measured under H_2_O_2_ deprivation conditions, and persulfidation of selected V-ATPase subunits was determined in control, Cd-, H_2_O_2_- and JA-treated cucumber plants. Using *Arabidopsis* mutants, three conserved Cys residues of VHA-A were analyzed to identify which of them are targeted by this modification. Finally, the signaling pathways induced by the two negative regulators of the pump, JA and cadmium, were compared.

## 2. Results

### 2.1. Regulation of the Vacuolar H^+^-ATPase by Jasmonic Acid

**Figure 1:**
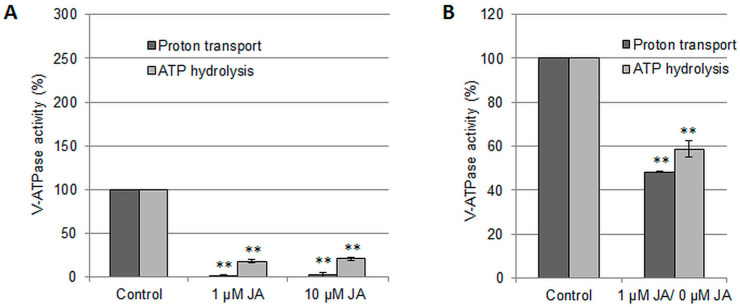
Effect of JA on ATP-dependent H^+^ transport and ATP hydrolysis in tonoplast vesicles isolated from cucumber roots. (**A**) Plants were treated with 1 μM JA or 10 μM JA for 24 h. (**B**) Plants were subjected to 1 μM JA for 24 h and then transferred to basic medium for an additional 24 h. For (**A**), the mean ATP-dependent proton transport of control samples was 0.65 ΔA_495_ min^−1^ mg^−1^ protein (100%), while the hydrolytic activity of V-ATPase reached a mean value of 165.01 μg Pi h^−1^ mg^−1^ protein (100%). In the pretreatment experiment (**B**), mean H^+^ translocation through tonoplast vesicles isolated from control plants was 0.40 ΔA_495_ min^−1^ mg^−1^ protein (100%), and ATP decomposition in the control sample achieved a mean value of 93.22 μg Pi h^−1^ mg^−1^ protein (100%). The data represents the means of 3 biological repetitions ± SE. Statistically significant differences (one-sample *t* test) between the control and treatments are marked as ** (*p* < 0.01).

To verify whether jasmonic acid is involved in the regulation of vacuolar H^+^-ATPase activity, cucumber seedlings were treated with 1 µM or 10 µM JA for 24 h, and ATP-dependent proton pumping and ATP hydrolysis were determined in tonoplast fractions isolated from the roots. Both JA concentrations inhibited enzyme activities similarly. Proton transport was drastically reduced to approximately 2%, whereas ATP hydrolysis decreased below 20% of the control level (Figure 1A). This suggests that JA functions as a molecule involved in the negative regulation of V-ATPase. Because the JA effect on V-ATPase was similar in the range of the tested hormone concentrations, we decided to focus on 1 µM JA in all subsequent analyses. Pretreatment of plants with 1 µM JA and their transfer to control medium for an additional 24 h indicated that the inhibitory effect of JA on the V-ATPase was not persistent but rather reversible, although the enzyme activity did not reach the initial level observed in control conditions (Figure 1B).

**Figure 2:**
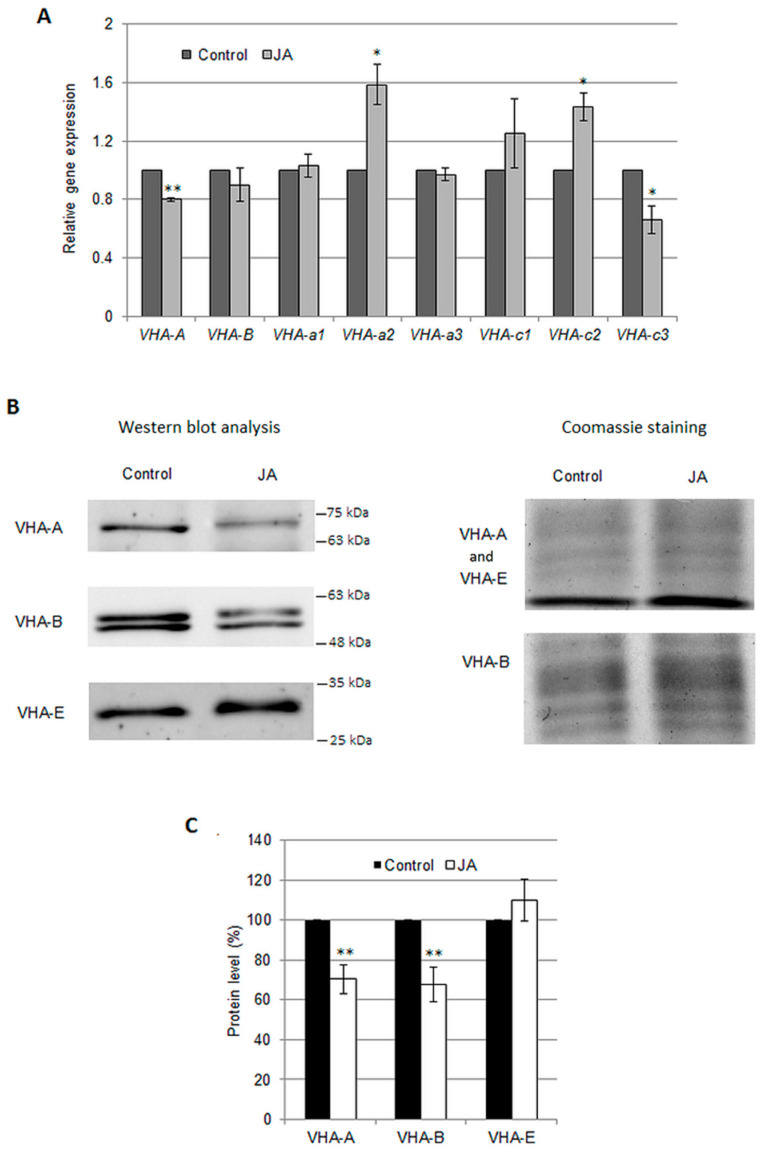
Expression of selected *VHA* genes (**A**) and protein levels of VHA-A, VHA-B, and VHA-E subunits (**B**,**C**) in *C. sativus* roots treated with 1 μM JA for 24 h. (**A**) qPCR analysis was performed using 4 reference genes (Table S1). The expression level of the control was normalized to 1. The data represent the means of 3 biological replicates ± SE. (**B**) Left panel—representative image of Western blot analysis of total protein extract from *C. sativus* roots with anti-VHA-A, anti-VHA-B, and anti-VHA-E antibodies. The numbers on the right represent the molecular weight marker bands. Right panel: -Coomassie staining of a part of the gel without proteins of interest, used as a protein loading control. (**C**) Average levels of VHA-A, VHA-B, and VHA-E proteins in *Cucumis* roots. The intensity of the Western blot signals was analyzed using ImageLab™. The results are presented as a % of the protein level measured under control conditions (100%). The data represents the means of 8 biological repetitions ± SE. Statistically significant differences (one-sample t test) between the control and treatments are marked as * (0.01 ≤ *p* < 0.05) or ** (*p* < 0.01).

To determine the molecular basis of the JA-dependent decrease in V-ATPase activity, the expression of the eight *VHA* genes, encoding four cucumber V-ATPase subunits, was analyzed. These subunits included VHA-A and VHA-B of the catalytic, cytosolic V_1_ sector, responsible for ATP hydrolysis, as well as VHA-a (a1, a2, and a3 isoforms) and VHA-c (c1, c2, and c3 isoforms) forming the membrane-bound proton pumping V_0_ sector (Figure 2A). It was observed that the levels of *VHA-A* and *VHA-c3* transcripts decreased by 20% and 34%, respectively, in JA-exposed plants. On the other hand, however, the expression of the *VHA-a2* and *VHA-c2* genes was visibly upregulated by JA (Figure 2A). Therefore, no coordinated changes in the expression of all the analyzed genes were found in response to JA. The results suggest that JA-induced modulation of V-ATPase activity was not closely related to the level of *VHA* gene expression.

In the next step, the protein level of selected proton pump subunits, including VHA-A, VHA-B, and VHA-E, was determined in the roots of control plants and plants treated with JA (Figure 2B,C). Interestingly, although *C. sativus* has only one gene encoding the VHA-B subunit [3,32], Western blot analysis showed the presence of two distinct bands corresponding to proteins of similar mass (Figure 2B); therefore, the cumulative signal from both of them was considered in the calculations. Jasmonic acid was found to decrease the protein levels of the A and B subunits in cucumber roots to approximately 70% of the control level, while the VHA-E protein level remained unaltered (Figure 2C). These results suggested that the hormone can reduce V-ATPase activity by lowering the protein amount of essential enzyme subunits, also in a manner unrelated to transcriptional regulation.

### 2.2. Involvement of H_2_S and H_2_O_2_ in the Regulation of Vacuolar H^+^-ATPase by Jasmonic Acid

**Figure 3:**
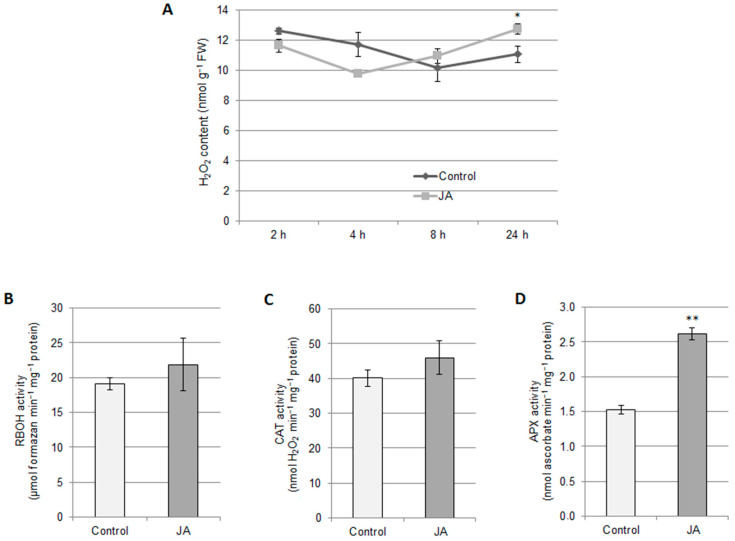
Effect of JA on the H_2_O_2_ level (**A**) and activity of H_2_O_2_ producing (**B**) or decomposing (**C**,**D**) enzymes in cucumber roots. (**A**) H_2_O_2_ content was measured in plants treated with 1 μM JA for 2, 4, 8, and 24 h. The data represent the means of 6 biological repetitions ± SE. Activity of the plasma membrane NADPH oxidase (**B**), catalase (**C**), and ascorbate peroxidase (**D**) was measured in plants exposed to 1 μM JA for 24 h. The data represents the means of 4 biological repetitions ± SE. Statistically significant differences (*t* test) are marked as * (0.01 ≤ *p* < 0.05) or ** (*p* < 0.01).

As we have demonstrated previously, the signaling molecules H_2_S and H_2_O_2_ control the V-ATPase activity in the roots of cucumber seedlings, acting as positive and negative regulators of this enzyme, respectively [31]. For this reason, the interrelationships between JA and both molecules were analyzed. Treatment of plants with JA for 24 h significantly increased the endogenous H_2_O_2_ level in roots to 115% of the control. In contrast, short-term exposure of cucumber seedlings to JA (2 h, 4 h, and 8 h) did not result in significant changes in the H_2_O_2_ content (Figure 3A). The observed increase in H_2_O_2_ level could be related to the action of H_2_O_2_-generating or decomposing enzymes. However, no changes in the activities of plasma membrane NADPH oxidase and catalase were found after plant treatment with JA for 24 h (Figure 3B,C). Under the same conditions, the activity of APX was stimulated, achieving approximately 170% compared to the control (Figure 3D).

**Figure 4:**
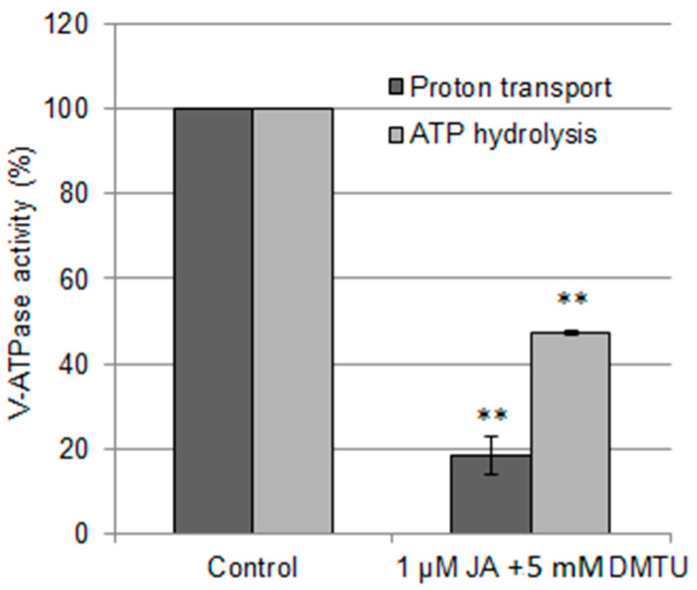
JA-dependent V-ATPase regulation under DMTU, an H_2_O_2_ scavenger. 1 μM JA with the addition of 5 mM DMTU was introduced to the growing medium for 24 h. The data represents the means of 3 biological repetitions ± SE. The mean ATP-dependent proton transport of control samples was 0.66 ΔA_495_ min^−1^ mg^−1^ protein (100%). The hydrolytic activity of V-ATPase reached a mean value of 163.56 μg Pi h^−1^ mg^−1^ protein in control samples (100%). Statistically significant differences (one-sample *t* test) between the control and treatments are marked as ** (*p* < 0.01).

The obtained results suggested that jasmonic acid can diminish the vacuolar proton pump action by enhancing the level of H_2_O_2,_ a well-known V-ATPase downregulator. To confirm this possibility, V-ATPase activity was determined in cucumber seedlings exposed to JA together with the H_2_O_2_ scavenger, 5 mM dimethylthiourea (DMTU). Although in these seedlings the enzyme activity is still decreased and achieved 18% and 47% of the control values for H^+^ transport and ATP hydrolysis, respectively (Figure 4), when compared to plants treated with JA alone (Figure 1A), the level of decreasing of both values was much lower. In this way, we observed a nearly 9-fold increase in H^+^ transport into tonoplast vesicles and an approximately 2,5-fold increase in ATP hydrolysis catalyzed by the pump in the presence of DMTU. Hence, lowering the H_2_O_2_ concentration during JA exposure markedly improved V-ATPase functioning by decreasing the JA effect.

**Figure 5:**
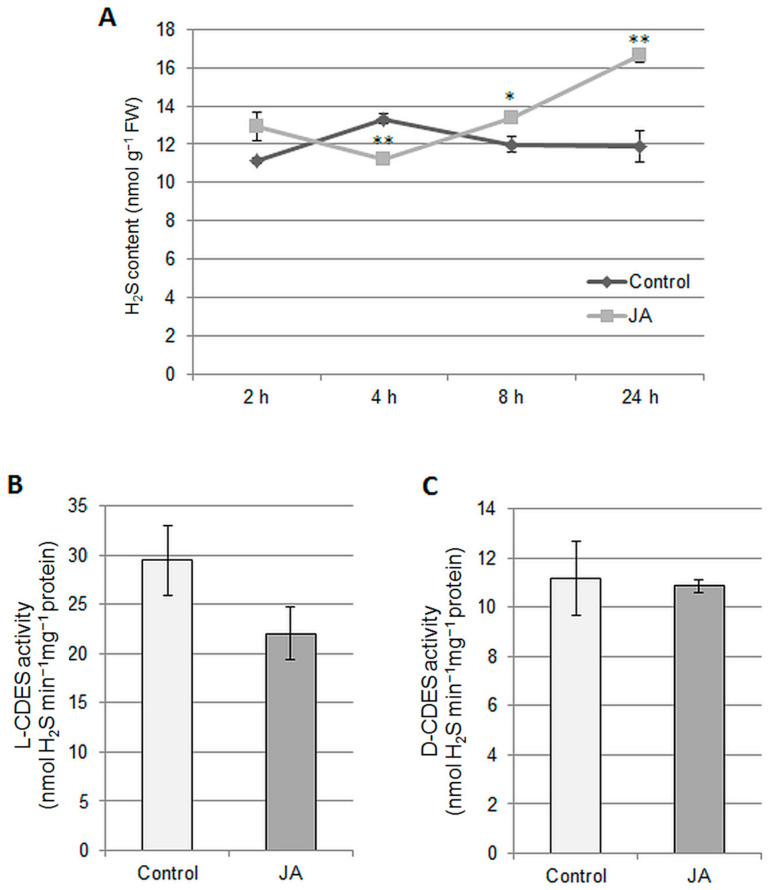
Effect of JA on the H_2_S content (**A**) and activity of H_2_S-generating enzymes (**B**,**C**) in *Cucumis* roots. (**A**) The H_2_S level was measured in plants treated with 1 μM JA for 2, 4, 8, and 24 h. The data represents the means of 6 biological repetitions ± SE. Statistically significant differences (t test) between the control and JA treatments are marked as * (0.01 ≤ *p* < 0.05) or ** (*p* < 0.01). The activities of L-cysteine desulfhydrase (**B**) and D-cysteine desulfhydrase (**C**) were measured in plants treated with 1 μM JA for 24 h. The data represent the means of 4 biological repetitions ± SE. No significant differences (*t* test, *p* < 0.05) were found.

In addition, we observed that the endogenous level of H_2_S fluctuated during the plant’s response to JA. An initial decrease in the H_2_S content, recorded after 4 h of plant exposure to the hormone, was followed by an increase above the control value. The highest level of H_2_S, 140% of the control, was observed after 24 h of JA treatment (Figure 5A). The results suggested that H_2_S may be another signaling molecule operating downstream in the JA-induced V-ATPase regulation pathway. For this reason, the JA effect on the activities of the main enzymes participating in H_2_S generation in the cytosol, L-cysteine desulfhydrase (L-CDES) and D-cysteine desulfhydrase (D-CDES), was determined. It was found that 24-h JA application did not significantly affect the action of both enzymes in cucumber roots (Figure 5B,C).

Based on the obtained data, it can be concluded that JA causes an increase in the H_2_O_2_ level in cucumber roots, which negatively affects the activity of V-ATPase. This regulation, however, does not involve the plasma membrane NADPH oxidase. Other H_2_O_2_-generating enzymes may participate in this process. Moreover, jasmonic acid increases the H_2_S content in the root tissues. The mechanism by which JA-dependent H_2_S production occurs has not been elucidated; however, it is independent of desulfhydrase activity.

### 2.3. Direct Effect of H_2_S on the Vacuolar H^+^-ATPase Activity and Persulfidation of Its Subunits

**Figure 6:**
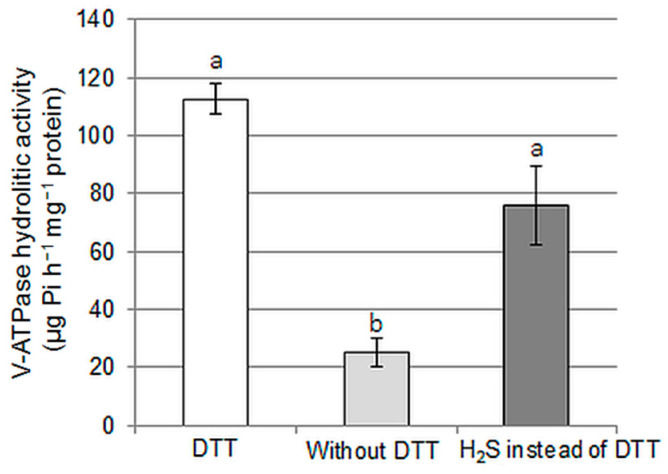
Modification of V-ATPase activity by H_2_S addition during tonoplast preparation. ATP hydrolysis was determined in the tonoplast vesicle fraction isolated from cucumber roots using a standard procedure (with the presence of DTT in all solutions) in the absence of DTT or buffers enriched with NaHS (H_2_S donor) instead of DTT. The DTT and H_2_S concentrations were equal, 1 mM or 5 mM, depending on the isolation step. The data represents the means of 3 biological repetitions ± SE. Homogeneous groups, according to Tukey’s test (*p* < 0.05) are marked with letters.

Our previous results [31] indicated that exogenously applied NaHS (H_2_S donor) stimulated the tonoplast proton pump in cucumber roots; however, the precise mode of H_2_S action has not yet been clarified. Therefore, in the current work, we tested the possibility of a direct effect of this signaling molecule on the V-ATPase. We compared the hydrolytic activity of V-ATPase in tonoplast vesicles isolated from cucumber roots in three different ways: using a standard procedure with the presence of DTT in all solutions, in the absence of DTT, or using buffers enriched with NaHS instead of DTT. The DTT and H_2_S concentrations were equal, 1 mM or 5 mM, depending on the isolation step. As shown in Figure 6, the presence of H_2_S in buffers increased the hydrolytic activity of the enzyme approximately threefold compared to that observed in vesicles isolated without DTT, suggesting that H_2_S protects V-ATPase from oxidation-dependent downregulation. At the same time, no significant differences were found between the enzyme activities measured using DTT or NaHS during isolation (Figure 6).

**Figure 7:**
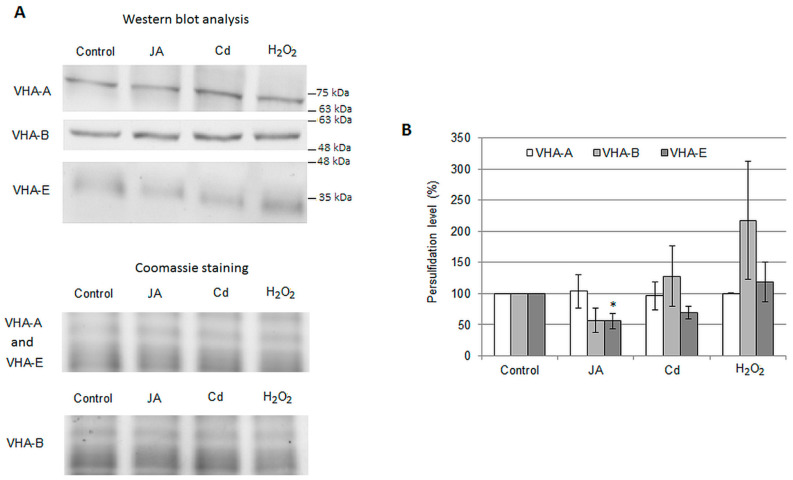
Persulfidation of the proton pump subunits. (**A**) Upper panel—representative image of Western blot analysis of VHA-A, VHA-B, and VHA-E subunits in the fraction of persulfidated proteins isolated from *Cucumis* roots exposed to 1 μM JA, 100 μM CdCl_2_, or 5 mM H_2_O_2_ for 24 h. Lower panel—Coomassie staining of a part of the gel without proteins of interest used as a protein loading control. (**B**) Average level of persulfidation of cucumber V-ATPase subunits A, B, and E treated with 1 μM JA, 100 μM CdCl_2_, or 5 mM H_2_O_2_. The intensity of the Western blot signals was analyzed using ImageLab™. The results are presented as the % of subunit persulfidation measured under control conditions (100%). Data represents the means of 3–5 biological repetitions ± SE. Statistically significant differences (one-sample t test) between the control and treatment are marked as * (0.01 ≤ *p* < 0.05).

The protective effect of H_2_S may be related to its antioxidant properties or to the persulfidation of the proton pump subunits. To gain insight into the mode of H_2_S action, we verified whether the proton pump undergoes persulfidation. It was found that all three V-ATPase subunits selected for the analysis, i.e., VHA-A, VHA-B, and VHA-E, were persulfidated in cucumber roots under control conditions (Figure 7A). Intriguingly, although two bands were visible after immunoblot analysis with anti-VHA-B antibodies (Figure 2B), only one of them, corresponding to the protein with a greater mass, was present among the persulfidated proteins (Figure 7A). In addition, the signal from persulfidated VHA-E appeared to be relatively weak, suggesting that only a small fraction of the VHA-E protein was modified (Figure 7A). Additionally, persulfidation of V-ATPase subunits was analyzed under various treatment conditions, which were found to increase H_2_S levels in *Cucumis* roots. These included 1 µM JA, 5 mM H_2_O_2_, and 100 µM CdCl_2_ [31]. No significant effect of 24 h treatment with JA, Cd, and H_2_O_2_ on the persulfidation of the A and B subunits was observed. In contrast, JA decreased the persulfidation of VHA-E (Figure 7B).

**Figure 8:**
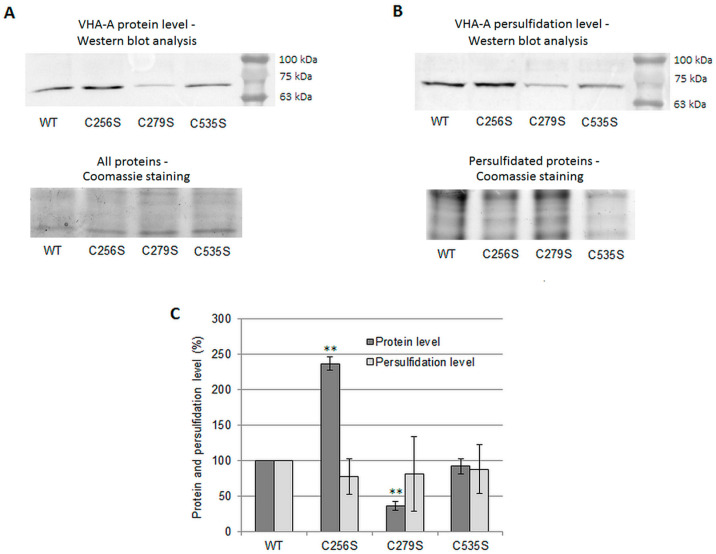
Persulfidation of the VHA-A subunit in *Arabidopsis thaliana* WT and mutants with substitution of a single Cys residue to Ser (C256S, C279S, and C535S). (**A**) Upper panel—representative image of Western blot analysis of total protein extract from *A. thaliana* leaves with anti-VHA-A antibodies; the numbers on the right represent the molecular weight marker overlay using ImageLab^TM^. Lower panel—Coomassie staining of a part of the gel without proteins of interest used as a protein loading control. (**B**) Upper panel—representative image of Western blot analysis of the VHA-A subunit in the fraction of persulfidated proteins isolated from A. thaliana leaves; the numbers on the right represent the molecular weight marker overlay using ImageLab^TM^. Lower panel—Coomassie staining of a part of the gel without proteins of interest used as a protein loading control. (**C**) Average level of VHA-A protein and its persulfidation in leaves of A. thaliana WT and mutants. The intensity of the Western blot signals was analyzed using ImageLab™. The results are presented as the % of protein level or persulfidation level measured in WT plants (100%). The data represents the means of 3–4 biological repetitions ± SE. Statistically significant differences (one-sample *t* test) between the control and treatments are marked as ** (*p* < 0.01).

The possible target of persulfidation in VHA-A may be conserved Cys residues, previously described as involved in V-ATPase redox regulation in other species [9]. Alignment of protein sequences of VHA-A originating from organisms from different branches of the tree of life confirmed their presence in *C. sativus*. These included Cys^256^, Cys^279^, and Cys^535^, according to the numbering for cucumber and *Arabidopsis* proteins (Figure S1). To verify which of the conserved Cys residues are persulfidated, leaves from wild-type *Arabidopsis thaliana* and three corresponding mutants created by Seidel et al. [33] were used for tag-switch analysis. In each of the mutants, one of the conserved Cys residues in VHA-A was substituted with Ser, C256S, C279S, or C535S, respectively. It was found that replacing Cys^256^ with Ser caused a more than twofold increase in the level of VHA-A protein, while in the C279S mutant, the amount of this subunit was three times lower than that in the leaves of WT *Arabidopsis*. No changes were observed in the protein level of VHA-A in C535S plants in comparison to the WT (Figure 8A,C). The determination of the persulfidation level indicated, however, that none of the mutants exhibited a significantly reduced level of this posttranslational modification within VHA-A (Figure 8B,C).

## 3. Discussion

### 3.1. Jasmonic Acid Treatment Reduces V-ATPase Activity in Cucumber Roots

Endogenous factors participating in signal transduction pathways leading to the regulation of the plant vacuolar H^+^-ATPase are not fully understood. Here we showed that jasmonic acid applied at 1 µM concentration, which is one of the lowest concentrations commonly used in plant research [27], has a strong, negative, and reversible effect on the V-ATPase function in the tonoplast of cucumber root cells (Figure 1A,B). The reduction in proton pump activity as a result of plant treatment with JA seems to be consistent with the known mechanism of JA action. This hormone is a well-known inhibitor of the growth of various plant organs [34], while V-ATPase is involved in turgor pressure regulation, nutrient storage, and vesicle trafficking, enabling cell growth [35,36].

On the other hand, the study of Brüx et al. [37] showed that the relationship between jasmonates and V-ATPase is more complex. The authors proved that genetically decreased V-ATPase activity in the *Arabidopsis det3* mutant is correlated with an increased concentration of OPDA (12-oxophytodienoic acid), an intermediate in the synthesis of JA. OPDA synthesis is stimulated as an element of the defense response provoked by altered functioning of the pump in the TGN/EE but not in the vacuole. Decreased V-ATPase activity in TGN/EE causes disturbances in vesicular transport and cellulose synthesis that are perceived as disruptions of cell wall integrity, comparable to an injury or infection. Interestingly, no increase in JA or JA-Ile content was observed in this study, and OPDA was suggested to induce inhibition of cell elongation via a pathway independent of the JA receptor. The authors pointed out that some of the physiological effects observed after JA or MeJA treatments may be independent of JA due to the well-documented positive feedback and increased OPDA content in the cell after JA application [37].

Contrary to the results of our studies conducted on cucumber roots, stimulation of V-ATPase hydrolytic activity was shown in leaves of *A. thaliana* [38] and *Hordeum vulgare* [39] treated with MeJA. Since MeJA is not active per se but is hydrolyzed to JA and then converted to JA-Ile [40], JA and MeJA should induce the same physiological responses. However, in both cases, a positive effect of the hormone on V-ATPase activity was noted during MeJA-induced leaf senescence [38,39], while cucumber roots exposed to JA do not show any typical macroscopic hallmarks of senescence, such as root browning or shrinking [41]. Moreover, JA removal restores V-ATPase activity (Figure 1B); therefore, root cells are still alive. The above findings suggest that jasmonates orchestrate V-ATPase activity depending on the plant’s developmental state and its current needs.

Interestingly, Ratajczak et al. [39] described upregulation of ATP hydrolysis after the hormone treatment; however, they were unable to demonstrate Bafilomycin A_1_-sensitive H^+^ translocation into tonoplast vesicles isolated from barley leaves incubated with MeJA. Thus, the authors supposed that MeJA increases vacuolar membrane permeability [39]. This hypothesis explains the much stronger JA effect on proton transport than on ATP hydrolysis catalyzed by the cucumber proton pump (Figure 1A,B).

### 3.2. Jasmonic Acid Decreases the Protein Level of V-ATPase Subunits

The activity of V-ATPase as a multi-subunit enzyme is complicated and regulated by many different mechanisms [2]. Numerous studies [38,42,43,44] have demonstrated uncoordinated transcription of *VHA* genes in response to different stimuli, suggesting that particular proton pump subunits play specific roles in enzyme regulation. In addition, it was shown that downregulation [45] or overexpression [46,47] of a single *VHA* gene affects V-ATPase activity. Thus, changing the level of one protein that builds the holoenzyme modulates its functioning. Our analysis showed that JA treatment induces a significant decrease in the amount of VHA-A and VHA-B (Figure 2B,C). This should be at least partially responsible for the observed V-ATPase downregulation, especially because these two subunits build the catalytic head of the enzyme [2]. A simultaneous decrease in V-ATPase activity and VHA-A and VHA-B content was also observed in the *Vigna unguiculate* hypocotyls under saline conditions [48].

At the molecular level, the JA action is based on the modulation of gene transcription [40]. However, in cucumber, only the VHA-A subunit seems to be regulated at the transcriptional level (Figure 2A). In contrast, VHA-B appears to be modulated posttranscriptionally, or alternatively, JA exposure could stimulate VHA-B degradation. Cadmium-induced VHA-A proteolysis has been found in *Arabidopsis* [49], while VHA-B degradation is correlated with the transition from C_3_ to CAM photosynthesis in *Mesembryanthemum crystallinum* [50]. The mechanism of enzymatic degradation may explain the VHA-B behavior in JA-treated plants, especially based on the appearance of additional polypeptides with molecular masses similar to VHA-B detected after treatment of barley leaf segments with MeJA [39].

However, in the current study, regardless of the JA application, two types of VHA-B were found (Figure 2B), differing in susceptibility to persulfidation (Figure 7A). Truncated variants of V-ATPase subunits, including VHA-B, have already been observed in a few organisms [50,51,52]. Moreover, in *Arabidopsis*, four transcripts of different lengths are formed as a result of the expression of a single *VHA-A* gene, which is related to the presence of alternative polyadenylation sites in the mRNA [53]. It was supposed that the production of different variants of VHA proteins might be a way of regulating holoenzyme stability [52].

Because some VHA subunits are encoded by several isogenes, it has been postulated that particular VHA isoforms may be incorporated into the holoenzyme depending on the development stage or environmental factors. This allows plants to flexibly adapt to changing conditions [43]. In cucumber, JA enhances the level of *VHA-a2* and *VHA-c2* mRNA (Figure 2A); therefore, it cannot be ruled out that hormone treatment supports the inclusion of VHA-a2 and VHA-c2 into the V-ATPase complex instead of other a and c isoforms, affecting enzyme activity. Similarly, in *Arabidopsis*, MeJA upregulates the transcription of *VHA-B2* and *VHA-E3* but not *VHA-B1*, *VHA-B3*, or *VHA-E2* [38,54]. 

### 3.3. H_2_O_2_ Participates in JA-Dependent V-ATPase Downregulation

Jasmonates are known to stimulate ROS production, especially H_2_O_2_. In many studies, JA-dependent H_2_O_2_ biosynthesis is coupled with enhanced RBOH action [27,55,56,57]. Although H_2_O_2_ content in cucumber roots also increased after JA treatment (Figure 3A), it was not correlated with changes in RBOH activity (Figure 3B), suggesting that a different H_2_O_2_-generating source was activated. On the other hand, in *Cucumis sativus*, such as in *Cucumis melo* cell lines [58], JA induces upregulation of APX (Figure 3D), responsible for H_2_O_2_ decomposition. It is assumed that APX, in contrast to CAT, is involved in the precise modulation of H_2_O_2_ level, especially during signal transduction [59]. Thus, cucumber seedlings maintain H_2_O_2_ levels under strict control, and in cucumber, as in other species [27,55,56], H_2_O_2_ can probably function as a messenger in jasmonate signaling. This thesis is supported by an experiment using DMTU, a H_2_O_2_ scavenger, which confirmed the involvement of H_2_O_2_ in the downregulation of V-ATPase observed after JA treatment (Figure 4). Furthermore, considering that DMTU only partially restores V-ATPase activity (Figure 4), JA appears to simultaneously induce several pathways that negatively regulate the proton pump.

### 3.4. H_2_S and Persulfidation Can Function as Elements of the JA Signaling Involved in V-ATPase Regulation

It is well established that there is a close relationship between the response to jasmonates and the transcriptional induction of sulfur assimilatory genes [60,61]. However, knowledge about the activation of hydrogen sulfide-generating enzymes in response to this hormone is still scarce. Therefore, upregulation of H_2_S levels has been shown in leaves of *Setaria italica* [62] and *Arabidopsis* [30,63] after JA or MeJA exposure, respectively. Similarly, H_2_S content in cucumber roots fluctuates after JA application (Figure 5A), suggesting that H_2_S can function as a secondary transmitter in the JA-dependent signaling network. In *Arabidopsis*, L-Cys desulfhydrase and D-Cys desulfhydrase have been identified as the main sources of H_2_S produced in response to MeJA [63], while in *C. sativus*, neither of the two enzymes was stimulated by JA (Figure 5B,C). Thus, in cucumber, other H_2_S-generating enzymes participate in this process or, alternatively, downregulation of H_2_S-assimilatory enzymes, such as O-acetylserine (thiol) lyase, occurs in response to the hormone.

The role of H_2_S in signal transduction is related to its ability to react with reactive oxygen and nitrogen species (antioxidant activity), to interact with metal ions, e.g., heme iron, or to persulfidate cysteine residues [22]. In the current work, we showed that in vitro H_2_S application has a direct positive impact on the proton pump activity (Figure 6), suggesting that persulfidation may modulate V-ATPase functioning. Furthermore, in cucumber, as in *Arabidopsis* [17,18], the A, B, and E V-ATPase subunits were detected in the fraction of persulfidated proteins isolated from plants grown under control conditions (Figure 7A). This confirms that endogenous H_2_S is able to induce this PTM of the proton pump and suggests that stimulation of V-ATPase activity after H_2_S donor application, observed in plants [31] as well as animals [64], is probably related to its persulfidation. 

The occurrence of persulfidation requires specific conditions and must be preceded by Cys oxidation to the -SOH form [20]. Therefore, we assumed that JA, Cd^2^+, or H_2_O_2_ exposure, by increasing both H_2_O_2_ and H_2_S contents in *Cucumis* roots (Figure 3 and Figure 5A, [31]), could enhance the level of V-ATPase modification by H_2_S. However, no changes in persulfidation of the A and B subunits were observed as a result of JA, Cd^2+^ or H_2_O_2_ application. In contrast, in VHA-E, JA treatment decreased the level of its modification (Figure 7A,B); therefore, in this way, the hormone may diminish the V-ATPase activity. The lack of persulfidation changes may suggest that H_2_S is produced in other subcellular locations, distant from V-ATPase. Moreover, analysis on *des1 Arabidopsis* plants, which have a lower level of H_2_S than WT, shows a similar number of persulfidated proteins. This clearly indicates that a change in total H_2_S content is not enough to modulate the persulfidation level of the V-ATPase, and additional factors must exist [17]. 

### 3.5. Persulfidation Level of VHA-A Does Not Change in Arabidopsis Mutants with a Substitution of Conserved Cys Residues within This Subunit

V-ATPase, such as many enzymes, requires a reducing reagent (DTT) to achieve maximum activity (Figure 6; [65]). The mechanism of pump regulation by redox potential, initially proposed for animals, involves intrasubunit disulfide swapping between three cysteine residues within VHA-A conserved in all eukaryotes (Figure S1); [33]). Inhibition of V-ATPase occurs by the S‒S bond formation between Cys^256^ and Cys^535^ (numeration for *Arabidopsis* protein), near the catalytic site of the enzyme, while activation is a result of the formation of a bridge between Cys^535^ and Cys^279^ [9]. However, more detailed analysis performed by Seidel et al. [33] revealed that, in *Arabidopsis*, H_2_O_2_-induced inhibition of the proton pump depends on only one cysteine residue in VHA-A, Cys^256^.

This amino acid appears to have unique properties since treatment of *Arabidopsis* cell culture with 20 mM H_2_O_2_ induces sulfenylation of all Cys residues in VHA-A, with the exception of this one [13,66]. Thus, Cys^256^, under very high H_2_O_2_, is still reduced or, more likely, easily undergoes overoxidation. Hence, downregulation of *Arabidopsis* V-ATPase by H_2_O_2_ may be a result of the sulfenylation (-SOH) of Cys^256^ or its further oxidation. We supposed that persulfidation of VHA-A occurs at the same Cys residue since H_2_S increased V-ATPase activity (Figure 6), and one of the well-described functions of persulfidation is the protection of proteins against irreversible modification by ROS [20]. 

However, studies using *Arabidopsis* transgenic lines with substitutions C256S, C279S, and C535S showed that neither of the tested Cys residues is the target of H_2_S (Figure 8B, C). This means that H_2_S can modify one of the other three Cys present in VHA-A or V-ATPase, which can require modification of several of the six Cys residues within the A subunit at the same time (Figure S1). In the last case, even if some of the conserved Cys are persulfidated, a single mutation might not result in a measurable decrease in the overall level of VHA-A modification by Western blot densitometry. 

Interestingly, although Seidel et al. [33] observed both reduced ATP hydrolysis activity in tonoplast fraction isolated from the C279S line as well as decreased H^+^-translocation in C256S and C535S plants, they found no changes in the level of the VHA-A protein between WT and mutants. In contrast, our studies indicated that C256S plants are characterized by higher and C279S plants by lower protein levels of the A subunit (Figure 7A). This suggests that substitution of Cys^256^ and Cys^279^ affects the stability/life time of VHA-A, for example, by changing protein folding, altering its architecture, or disrupting the oxPTMs pattern [67]. On the other hand, the above results confirm that the activity of V-ATPase present in the vacuolar membrane does not seem to be directly correlated with the VHA-A protein level and persulfidation status, as well as with plant growth. This point of view, however, requires further research. 

### 3.6. The Mechanisms of V-ATPase Downregulation by JA and Cd Are Different

The cucumber response to JA shows some features in common with the cadmium stress response (Table S2). Both factors diminish V-ATPase activity and increase the contents of H_2_O_2_ and H_2_S in cucumber roots (Figure 1A,B, Figure 3A and Figure 5A, [31]). Moreover, the H_2_O_2_ scavenger DMTU restores V-ATPase activity in JA- and Cd-treated cucumber seedlings (Figure 4; [31]). This led us to hypothesize that JA may be involved in the Cd-induced downregulation of the proton pump.

A detailed study, however, contradicts the significant participation of JA in the Cd-induced signaling pathway involved in V-ATPase regulation in cucumber roots. Under cadmium stress, the synthesis of H_2_S is positively regulated by H_2_O_2_, and an increase in H_2_O_2_ level precedes an increase in H_2_S production [31]. In contrast, after JA treatment, H_2_S content changes more rapidly than H_2_O_2_ (Figure 3A and Figure 5A). Moreover, the sources of H_2_S production in response to JA and Cd seem to be different since JA does not affect the activity of L-Cys and D-Cys desulfhydrases (Figure 5B,C), while Cd stimulates them [31]. One of the reasons for the JA-induced V-ATPase downregulation is the reduction in the amount of VHA-A and VHA-B subunits (Figure 2B,C), whereas Cd increases the VHA-A level and only slightly decreases the VHA-B amount (Figure S2). Similarly, JA decreases VHA-E persulfidation, while Cd does not provoke any changes in the persulfidation level of V-ATPase subunits (Figure 7B). Although both JA and Cd decrease V-ATPase activity in cucumber, and some studies postulate JA involvement in the Cd stress response [68,69,70,71,72,73], the molecular basis of V-ATPase downregulation by these two factors appears to be different. 

## 4. Materials and Methods

### 4.1. The Plant Material, Growth Conditions and Treatments

Seeds of *Cucumis sativus* cv. Wisconsin were germinated at 25 °C in darkness for 2 days. Seedlings were grown hydroponically on a basic medium containing 1.7 mM Ca(NO_3_)_2_, 1.7 mM KNO_3_, 0.33 mM MgSO_4_, 0.33 mM KH_2_PO_4_, 25 μM ferric citrate, 3.3 μM MnSO_4_, 1.7 μM H_3_BO_3_, 0.3 μM CuSO_4_, 17 nM Na_2_MoO_4_, and 3 nM ZnSO_4_ (pH 6.2) under 180 μmol photons m^−2^s^−1^ of light with a 16 h (25 °C)/8 h (22 °C) light/dark regime and constant 70% relative humidity. After 5 days, cucumber seedlings were transferred for the next 24 h to fresh basic medium (pH 5.5, control) or basic medium (pH 5.5) with the addition of the following compounds: 1 µM JA, 10 µM JA, 100 μM CdCl_2_, 5 mM H_2_O_2_, or 5 mM dimethylthiourea (DMTU, H_2_O_2_ scavenger) [74,75]. In pretreatment experiments, plants were transferred to fresh media twice, after 4 and 5 days. The cucumber roots were collected after 6 days of growth. 

Seeds of *Arabidopsis thaliana* (Col-0) and corresponding *VHA-A* mutants were kindly provided by Prof. Karin Schumacher (Universität Heidelberg). Three transgenic lines (named C256S, C279S, and C535S) with a single substitution of the Cys residue with Ser (Cys^256^, Cys^279^, or Cys^535^, respectively) have been previously characterized [33]. Sterilized *Arabidopsis* seeds were soaked in distilled water at 4 °C. After 24 h, seeds were suspended in water, sown onto pots filled with Compo Sana Universal substrate (Compo, Poznań, Poland), and transferred to a growth chamber (16 h of light with an intensity of 120 μmol photons m^−2^s^−1^ at 20 °C and 8 h of night at 18 °C). After germination, only one plant was left per pot. *Arabidopsis* seedlings were watered once a week with tap water. The leaves were collected after 40 days of cultivation.

Most of the reagents used in this study were purchased from Pol-Aura (Morąg, Poland) and Sigma-Aldrich (St. Louis, MO, USA). 

### 4.2. Isolation of Membrane Fractions

Highly purified tonoplast vesicles were isolated from cucumber roots, according to Kabała and Kłobus [76]. A discontinuous sucrose density gradient consisting of 20, 28, 32, and 42% (*w*/*w*) sucrose was used. The third gradient fraction was tonoplast-enriched. 

Highly purified plasma membrane vesicles were isolated from cucumber roots according to Larsson [77], as modified by Kłobus [78]. A 6.2% two-phase system containing PEG (polyethylene glycol) 3350 and dextran T500 was used. The upper phase was plasma membrane-enriched.

### 4.3. Content of Signaling Molecules

The H_2_O_2_ content in cucumber roots was quantified according to Velikova et al. [79], as modified by Kabała et al. [31]. The procedure included the oxidation of potassium iodide (KI) by H_2_O_2_ and measuring the absorbance of the reaction product, triiodide (I_3_^−^), at 390 nm. The level of H_2_O_2_ was calculated based on a standard curve and expressed in nmol g^−1^ fresh weight (FW).

The H_2_S level was determined by the methylene blue (MB) method according to Li [80], as modified by Kabała et al. [31]. Briefly, tissue was ground with extraction buffer containing Zn(CH_3_CO_2_)_2_, and H_2_S was fixed in a zinc acetate trap. To liberate H_2_S from the zinc sulfide complex, strong acidic conditions were used. After incubation with FeCl_3_ and N,N-dimethyl-p-phenylenediamine (DMPD) dihydrochloride, the absorbance of the formed MB was measured at 670 nm. The content of H_2_S in nmol g^−1^ FW was quantified using the MB extinction coefficient ε = 9.5 × 10^4^ M^−1^ cm^−1^.

### 4.4. Determination of Enzyme Activities

The hydrolytic activity of V-ATPase (EC 3.6.3.14) was determined in tonoplast fractions according to Gallagher and Leonard [81], as described by Kabała et al. [65], based on the amount of inorganic phosphate released during 30 min of reaction as a result of ATP breakdown. Inorganic phosphate was assayed according to Ames [82], and its concentration was determined using a standard curve. The V-ATPase activity was expressed as vanadate-, azide-insensitive and nitrate-sensitive ATPase activity and calculated as μg Pi h^−1^ mg^−1^protein. ATP-dependent proton pumping was monitored in tonoplast vesicles spectrophotometrically for 3 min immediately after substrate addition as a drop in absorbance of acridine orange at 495 nm, according to Kabała et al. [65]. This activity was expressed as ΔA_495_ min^−1^ mg^−1^ protein.

The activity of NADPH oxidase (RBOH, EC 1.6.3.1) was determined in plasma membrane fractions according to Sagi and Fluhr [83] as described by Jakubowska et al. [84] using NADPH as an electron donor and 2,3-bis(2-methoxy-4-nitro-5-sulfophenyl)-2H-tetrazolium-5-carboxanilide (XTT) sodium salt. Its conversion to formazan by the O_2_^.-^ radical, generated by NADPH oxidase, was assayed at 470 nm in the presence and absence of 50 units of Cu/Zn superoxide dismutase. Measurement was performed for 3 min, directly after XTT addition to the reaction mixture. The formazan extinction coefficient ε = 2.16 × 10^4^ M^−1^ cm^−1^ was taken for calculation, and enzyme activity was presented as μmol formazan min^−1^ mg^−1^ protein.

The activities of L-cysteine desulfhydrase (L-CDES, EC 4.4.1.1) and D-cysteine desulfhydrase (D-CDES, 4.4.1.15) were determined in the cytoplasmic fraction isolated from cucumber roots according to Siegel [85] as modified by Kabała et al. [31] using L-cysteine or D-cysteine, respectively, as substrates. The H_2_S released in the enzymatic reaction was measured by the MB method. The level of MB was quantified based on a standard curve using Na_2_S as a standard. The activity was expressed as nmol of H_2_S min^−1^ mg^−1^ of protein.

The activities of catalase (CAT, EC 1.11.1.6) and ascorbate peroxidase (APX, EC 1.11.1.11) were determined in the cytoplasmic fraction isolated from cucumber roots according to Weisany et al. [86]. CAT activity was measured by monitoring the decrease in H_2_O_2_ absorbance at 240 nm as a result of its decomposition by the enzyme, using ε = 43.6 × 10^−3^ M^−1^ cm^−1^. Its activity was calculated as the μmoles of decomposed H_2_O_2_ min^−1^ mg^−1^ protein. The APX-dependent oxidation of ascorbic acid was assayed by measuring the drop in absorbance of ascorbate at 290 nm, assuming an absorption coefficient of 2.8 × 10^−3^ M^−1^ cm^−1^. The enzyme activity was presented as μmol oxidized ascorbate min^−1^ mg^−1^ protein.

### 4.5. Gene Expression Analysis

Total RNA was isolated using EXTRAzol (BLIRT, Gdańsk, Poland) according to the manufacturer’s instructions. 2 µg of total RNA were taken for digestion with RNase-free DNase I (Thermo Fisher Scientific, Waltham, MA, USA), followed by cDNA synthesis with a High-Capacity cDNA Reverse Transcription Kit (Applied Biosystems, Foster City, CA, USA). The cDNA was diluted eight times and used as a template in qPCR. The expression of selected cucumber V-ATPase-encoding genes (*VHA-A*, *VHA-B*, *VHA-a1*, *VHA-a2*, *VHA-a3*, *VHA-c1*, *VHA-c2*, *VHA-c3*) and reference genes (actin, clathrin adaptor complex subunit, elongation factor 1-alpha and tonoplast intrinsic protein 41-like) was analyzed. The reaction was performed with a Real-Time 2 × PCR Master Mix SYBR kit (A&A Biotechnology, Gdańsk, Poland) using a LightCycler 480 system (Roche, Basel, Switzerland). The primers are listed in Table S1. The following qPCR conditions were applied: predenaturation at 95 °C for 30 s; 40 cycles of denaturation at 95 °C for 10 s; annealing at 58 °C (for *CsVHA-A*, *B*, *c2*, *c3*, *a1*, *a3*); 60 °C (for *CsVHA-c1*) or 66 °C (for *CsVHA-a2*) for 10 s; extension at 72 °C for 12 s; 15 s of final melting at 65 °C (for *CsVHA-A*, *B*, *a1*, *a3*, *c2*, *c3*); 68 °C (for *CsVHA-c1*) or 72 °C (for *CsVHA-a2*); and cooling (30 s/40 °C). Data analysis was performed with LightCycler software 4.1 (Roche, Basel, Switzerland). Precise information about the identification and characterization of *C. sativus* reference genes and V-ATPase genes has been described in Migocka and Papierniak [87] and Kabała et al. [31,32], respectively.

### 4.6. Determination of Protein Level

The Bradford protein assay [88], with BSA as a standard, was used to determine the protein level in all samples.

### 4.7. Preparation of Protein Extract

Proteins were extracted according to Jurado-Flores et al. [18], with some modifications. Plant material (cucumber roots or *Arabidopsis* leaves) was ground under liquid nitrogen to a fine powder. Frozen tissue (500 mg) was mixed by inversion with 400 μL of extraction buffer composed of 50 mM Tris-HCl (pH 7.5), 150 mM NaCl, 1 mM EDTA, 1 mM phenylmethylsulfonyl fluoride, and 1× protease inhibitor cocktail (cOmplete, Roche, Basel, Switzerland). The sample was centrifuged at 5000 rpm for 5 min at 4 °C. The supernatant was collected and centrifuged again. The procedure was repeated several times until a clear solution was obtained.

### 4.8. Purification of Persulfidated Proteins Using the Tag-Switch Method

The tag-switch technique was performed as described by Aroca et al. [17], with some modifications. To the protein extract (350 μg of total protein), ice-cold acetone and TCA were added in a 1:8:1 volume ratio. The sample was incubated at −20 °C for 1 h for protein precipitation. The protein pellet was dissolved in 200 μL of 50 mM Tris-HCl (pH 8.0) with 2.5% SDS, mixed with 50 μL of 250 mM methylsulfonyl benzothiazole solution (prepared in tetrahydrofuran), and incubated at 37 °C for 1 h with shaking at 550 rpm. Proteins were precipitated a second time overnight with five volumes of 100% ice-cold acetone at −20 °C.

The next day, the pellet was resuspended in 50 mM Tris-HCl (pH 8.0) with 2.5% SDS and 20 mM CN-biotin (biotin-linked cyanoacetate), incubated at 37 °C for 4 h with shaking (550 rpm), and precipitated a third time (overnight, −20 °C, 100% acetone, 1:5 ratio). The obtained proteins were mixed with 1 mL of 50 mM Tris-HCl (pH 8.0) containing 150 mM NaCl. To purify the persulfidated (biotin-labeled) proteins, the solution was incubated with 75 μL of Sera-Mag Magnetic Streptavidin-Coated Particles (GE HealthCare, Chicago, IL, USA) for 1 h with slow rotation (10 rpm). Then, the streptavidin beads were washed five times with Tris-HCl (pH 8.0) containing 600 mM NaCl and 1 mM EDTA. Bound proteins were eluted by incubation of beads with 6 M urea, 2 M thiourea, 30 mM CN-biotin, 100 mM NaCl, 50 mM NaH_2_PO_4_, and 2% SDS for 15 min at room temperature, followed by 15 min at 96 °C. The sample was mixed with ice-cold acetone and TCA (1:8:1 volume ratio) and kept overnight at −20 °C. The protein pellet was dissolved in 12 µL of extraction buffer and used for immunoblotting.

### 4.9. Western Blot and Coomassie Staining

A total of 9 µL of sample from the tag-switch procedure (persulfidation analysis) or a sample volume containing 10 µg of total protein (protein level analysis) were mixed with Laemmli buffer and denatured. Proteins were separated by electrophoresis using a 4% stacking and 10% separating polyacrylamide gel with the BioBLU Prestained Protein Ladder (Bio-Rad, Hercules, CA, USA) as a molecular weight marker. After SDS‒PAGE, the gel was cut into two parts: the part containing proteins smaller than 25 kDa (without proteins of interest) was used for Coomassie Brilliant Blue R-250 staining, and the remaining part was used for electrophoretic transfer.

After transfer, the membrane was blocked with 5% milk in phosphate-buffered saline with 0.1% (*v*/*v*) Tween 20 for 1 h, followed by overnight incubation at 4 °C with polyclonal antibodies against V-ATPase subunits A, B, or E (product numbers: AS09 467, AS14 2775, and AS07 213, respectively, Agrisera, Vännäs, Sweden). Primary antibodies were diluted 1:5000 or, in the case of *Arabidopsis* VHA-A detection, 1:7500. After overnight incubation, secondary antibodies conjugated with horseradish peroxidase (Goat Anti-Rabbit IgG (H+L)-HRP Conjugate, Bio-Rad, Hercules, CA, USA), diluted 1:10,000, were applied for 1 h. V-ATPase subunits were detected with Amersham ECL Select Western Blotting Detection Reagent (Cytiva, Marlborough, CT, USA) according to the manufacturer’s instructions. Blots and Coomassie staining gels were captured using ChemiDoc Imaging Systems (Bio-Rad, Hercules, CA, USA).

### 4.10. Quantification of the Protein Level of V-ATPase Subunits and Their Level of Persulfidation

Photos of blots and Coomassie staining gels were analyzed using Image Lab software version 5.2.1 (Bio-Rad, Hercules, CA, USA). Then, 100% was assumed to be the intensity of the signal measured under control conditions (experiments carried out on cucumber) or in wild-type *Arabidopsis* plants. To calculate the persulfidation of V-ATPase subunits, three factors were considered:

- intensity of the bands of the pre-tag-switch trials in the immunoblots (protein level of the sample before the tag-switch procedure compared to the control or wild type)
- Coomassie staining (protein amount in 9 µL of sample after the tag-switch procedure)
- intensity of the signal of samples after the tag-switch procedure in the immunoblots (persulfidated proteins).

### 4.11. Databases and Bioinformatics Tools

Amino acid sequences of the V-ATPase VHA-A subunit from different species were downloaded from UniProt [89] and aligned using Clustal Omega [90].

### 4.12. Statistical Analysis

To verify the normality of the data, the Shapiro-Wilk test was used (*p* > 0.05). The results expressed as % of the control were analyzed with a one-sample t test (*p* < 0.05). The homoscedasticity of the data was controlled with Brown-Forsythe tests. For two-group comparisons, the independent sample *t* test was performed (*p* < 0.05). If more than two groups of data were compared, one-way ANOVA was used (*p* < 0.05). Tukey’s test was chosen for posthoc analysis (*p* < 0.05). All statistical analyses were performed using Statistica version 13.3 (TIBCO Software Inc., Palo Alto, CA, USA). The results are presented as the means of a minimum of three biologically independent replicates’ ± standard error (SE).

## 5. Conclusions

**Figure 9:**
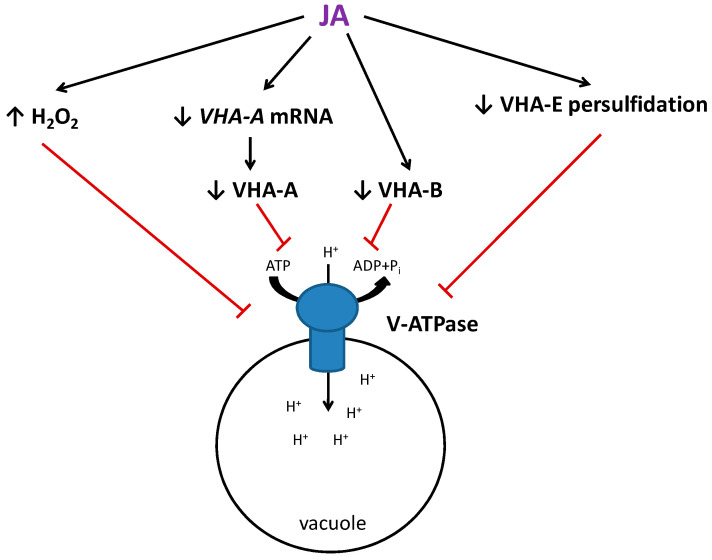
Mechanisms of V-ATPase downregulation by JA in cucumber roots.

In this study, jasmonic acid was found to be a downregulator of cucumber vacuolar H^+^-ATPase, and the mechanism of JA-dependent proton pump regulation has been proposed (Figure 9). We showed that the action of JA is related to the decrease in the protein amount of two important V-ATPase subunits, VHA-A and VHA-B, which build the catalytic head of the enzyme. Moreover, we demonstrated that H_2_O_2_, a well-known suppressor of V-ATPase, functions as a downstream component of the JA-induced signaling pathway, controlling enzyme activity. We also found that *Cucumis* V-ATPase subunits undergo persulfidation, and JA treatment decreases the persulfidation level of the VHA-E subunit. The comparison of signaling pathways induced by two repressors of the pump, JA and cadmium, revealed that multiple pathways are involved in the V-ATPase downregulation in cucumber roots (Table S2).

The decreased V-ATPase activity observed in this study may result in inhibition of central vacuole enlargement. This effect, together with the reduced activity of the plasma membrane proton pump, is responsible for limiting the growth of cucumber seedlings treated with jasmonic acid [91].

## Data Availability

The data presented are available in this manuscript and the Supplementary Materials.

## Supplementary Materials

The following supporting information can be downloaded at: https://www.mdpi.com/article/10.3390/ijms241813896/s1. References [92,93] are cited in the supplementary materials.
