# Supplementary material for: Regulation of V-ATPase by Jasmonic Acid: Possible Role of Persulfidation
Source: Int J Mol Sci. 2023 Sep 9;24(18):13896. doi: 10.3390/ijms241813896 (PMC10531226; doi:10.3390/ijms241813896)
Supplement: Supplementary file 1 [file ijms-24-13896-s001.zip › ijms-2558754-supplementary.pdf]

Supplementary material

Regulation of V-ATPase by jasmonic acid: Possible role of persulfidation

Magdalena Zboińska[1,2], Luis C. Romero[2], Cecilia Gotor[2], Katarzyna Kabała[1*]

[1]Department of Plant Molecular Physiology, Faculty of Biological Sciences, University of Wrocław, Kanonia 6/8, 50-328 Wrocław, Poland

[2]Instituto de Bioquímica Vegetal y Fotosíntesis, Consejo Superior de Investigaciones Científicas and Universidad de Sevilla, C. Américo Vespucio, 49, 41092 Sevilla, Spain

*Corresponding author: katarzyna.kabala@uwr.edu.pl

magdalena.zboinska@uwr.edu.pl

lromero@ibvf.csic.es

gotor@ibvf.csic.es

Table S1. The list of primers used in real-time quantitative PCRs.

Gene	Encoded protein	Primers sequences	Accession number
ACT	actin	**FP**: 5'CCGTTCTGTCCCTCTACGCTAGTG3'<br>**RP**: 5'GGAACTGCTCTTTGCACTCTCGAG3'	AB010922
CACS	clathrin adaptor complex subunit	**FP**: 5'TGGGAAGATTCTTATGAAGTGC3'<br>**RP**: 5'CTCGTCAAATTTACACATTGGT3'	GW881874
EF1	elongation factor 1α	**FP**: 5'ACTTTATCAAGAACATGATTAC3'<br>**RP**: 5'TTCCTTCACAATTTCATC3'	EF446145
TIP41	tonoplast intrinsic protein 41-like (PP2A phosphatase activator)	**FP**: 5' CAACAGGTGATATTGGATTATGATTATAC3'<br>**RP**: 5' GCCAGCTCATCCTCATATAAG3'	GW881871
VHA-A	V-ATPase subunit A	**FP:** 5'GTACTTCAGAGATATGGGATACAA3'<br>**RP**: 5'CGCTCATAGAAGGAGGCTAAA3'	JK998661
VHA-B	V-ATPase subunit B	**FP**: 5'GGTCAAGTCCTGGAAGTG3'<br>**RP**: 5'GGACCATTATCAATGGGCT3'	JZ971259
VHA-a1	V-ATPase subunit a1	**FP:** 5'GGTTTCTCTTTCGTTTCCGGTAA3'<br>**RP:** 5'TCTAAATTGAAGGATGCCAAGC3'	JZ971260
VHA-a2	V-ATPase subunit a2	**FP:** 5'AAGTTTGACATTAGGTTATTGGC3'<br>**RP:** 5'GGAAGACTCAGTAATGAAGGTAG3'	JZ971261
VHA-a3	V-ATPase subunit a3	**FP**: 5'GGAAATTGCCATTGTTCGT3'<br>**RP:** 5'TCATCACTTACAAAGACATTGGT3'	JZ971262
VHA-c1	V-ATPase subunit c1	**FP:** 5'AGTTACAATTACATACTCAACCTTT3'<br>**RP:** 5'CTTAGAGTCTTCGAACGTATCC3'	JK998658
VHA-c2	V-ATPase subunit c2	**FP:** 5'GGACCCAATCGTATTATTCTAAAGT3'<br>**RP**: 5'TTTCTGGGTTCAACGGTG3'	JK998659
VHA-c3	V-ATPase subunit c3	**FP:** 5'AGATACTCAAATTCATAAATTACGTTGAT3'<br>**RP:** 5'ACATTAACAATGACAAATGGTCC3'	JK998660

FP - forward primer
RP - reverse prime

Table S2. Comparison of the effects of jasmonic acid (JA) and cadmium (Cd) on V-ATPase and the level of signaling molecules in cucumber roots. Plants were treated with 1 µM JA or 100 µM $CdCl_2$ for 24 h. The data comes from current work and from the references listed in the table.

		JA	Cd	References
**V-ATPase activity**		↓	↓	**31,92**
**mRNA level of selected *VHA* genes**	*VHA-A*	↓	-	**31,92**
	VHA-B	-	↑	**31,92**
	VHA-a1	-	-	**31,92**
	VHA-a2	↑	↑	**31,92**
	VHA-a3	-	↓	**31,92**
	VHA-c1	-	↓	**31,92**
	VHA-c2	↑	↑	**31,92**
	VHA-c3	↓	-	**31,92**
**protein level of selected VHA subunits**	VHA-A	↓	-	**92**
	VHA-B	↓	-	**92**
	VHA-E	-	-	**92**
**H_2O_2content**		↑	↑	**31,92**
**reducing the level of H_2O_2 restores V-ATPase activity**		**yes**	**yes**	**31,92**
**activity of H_2O_2 producing and decomposing enzymes**	RBOH	-	-	**92**
	APX	↑	↓	**92**
	CAT	-	-	**92**
**H_2S content**		↑	↑	**31,92**
**activity of H_2S producing and decomposing enzymes**	L-CDES	-	↑	**31,92**
	D-CDES	-	↑	**31,92**
	OAS-TL	**nd**	-	**31**
**an increase in H_2O_2 level precedes an increase in H_2S content**		**no**	**yes**	**31,92**
**persulfidation of selected VHA subunits**	VHA-A	-	-	**92**
	VHA-B	-	-	**92**
	VHA-E	↓	-	**92**
**NO content**		↑	↓	**92,93**

↑ = increase; ↓ = decrease; - = no effect; nd = not determine

VHA-A

```
Saccharomyces      MAGAIENARKEIKRISLEDHAESEYGAIYSVSGPVVIAENMIGCAMYELVKVGHDNLVGE      60
Cucumis            ----MPSVYGDRLTTFEDSEKESEYGYVRKVSGPVVVADGMGGAAMYELVRVGHDNLIGE      56
Arabidopsis        ----MPAFYGGKLTTFEDDEKESEYGYVRKVSGPVVVADGMAGAAMYELVRVGHDNLIGE      56
Homo               -------MDFSKLPKILDEDKESTFGYVHGVSGPVVTACDMAGAAMYELVRVGHSELVGE      53
Drosophila_A1      ---------MSNLRKFKDEERESEYGRVYAVSGPVVTAEAMSGSAMYELVRVGYYELVGE      51
Drosophila_A2      ---------MSNLKRFDDEERESKYGRVFAVSGPVVTAEAMSGSAMYELVRVGYYELVGE      51
                                    :.. ** :* :  ****** *  * *.******:**: :*:**

Saccharomyces      VIRIDGDKATIQVYEETAGLTVGDPVLRTGKPLSVELGPGLMETIYDGIQRPLKAIKEES      120
Cucumis            IIRLEGDSATIQVYEETAGLMVNDPVLRTRKPLSVELGPGILGNIFDGIQRPLKTIAKRS      116
Arabidopsis        IIRLEGDSATIQVYEETAGLTVNDPVLRTHKPLSVELGPGILGNIFDGIQRPLKTIARIS      116
Homo               IIRLEGDMATIQVYEETSGVSVGDPVLRTGKPLSVELGPGIMGAIFDGIQRPLSDISSQT      113
Drosophila_A1      IIRLEGDMATIQVYEETSGVTVGDPVLRTGKPLSVELGPGIMGSIFDGIQRPLRDIGVMT      111
Drosophila_A2      IIRLEGDMATIQVYEETSGVTVGDPVLRTGKPLSVELGPGIMGSIFDGIQRPLKDINELT      111
                   :**::** *********:*: *.****** **********::  *:*******  *   :

Saccharomyces      QSIYIPRGIDTPALDRTIKWQFTPG-KFQVGDHISGGDIYGSVFENSLISSHKILLPPRS      179
Cucumis            GDVYIPRGVAVPALDKDTLWDFQPT-KIGEGDLLTGGDLYATVFENSLM-EHRIALPPEA      174
Arabidopsis        GDVYIPRGVSVPALDKDCLWEFQPN-KFVEGDTITGGDLYATVFENTLM-NHLVALPPDA      174
Homo               QSIYIPRGVNVSALSRDIKWDFTPCKNLRVGSHITGGDIYGIVSENSLI-KHKIMLPPRN      172
Drosophila_A1      NSIYIPKGVNTTALSRSEMWEFNPL-NVRVGSHITGGDLYGVVHENTLV-KQRMIVAPRA      169
Drosophila_A2      ESIYIPKGVNVPSLSRVASWEFNPL-NVKVGSHITGGDLYGLVHENTLV-KHKMIVNPRA      169
                    .:***:*: . :*.:   *:* *  :.  *. ::***:*. * **:*: .: : : *

Saccharomyces      RGTITWIAPAGEYTLDEKILEVEFDGKKSDFTLYHTWPVRVPRPVTEKLSADYPLLTGQR      239
Cucumis            MGKVTYIAAPGQYSLKDTVLELEFQGVRKQFTMLQTWPVRTPRPVASKLAADTPLLTGQR      234
Arabidopsis        MGKITYIAPAGQYSLKDTVIELEFQGIKKSYTMLQSWPVRTPRPVASKLAADTPLLTGQR      234
Homo               RGTVTYIAPPGNYDTSDVVLELEFEGVKEKFTMVQVWPVRQVRPVTEKLPANHPLLTGQR      232
Drosophila_A1      KGTVRYIAPAGNYNLEDIVLETEFDGEITKHTMLQVWPVRQPRPVTEKLPANHPLFTGQR      229
Drosophila_A2      KGTVRYIAPSGNYKVDDVVLETEFDGEITKHTMLQVWPVRQPRPVTEKLPANHPLLTGQR      229
                    *.: :**  *:*  .: ::* **:*   ..*: : ****  ***:.** *: **:****

Saccharomyces      VLDALFPCVQGGTTCIPGAFGCGKTVISQSLSKYSNSDAIIYVGCGERGNEMAEVLMEFP      299
Cucumis            VLDALFPSVLGGTCAIPGAFGCGKTVISQALSKYSNSDTVVYVGCGERGNEMAEVLMDFP      294
Arabidopsis        VLDALFPSVLGGTCAIPGAFGCGKTVISQALSKYSNSDAVVYVGCGERGNEMAEVLMDFP      294
Homo               VLDALFPCVQGGTTAIPGAFGCGKTVISQSLSKYSNSDVIIYVGCGERGNEMSEVLRDFP      292
Drosophila_A1      VLDSLFPCVQGGTTAIPGAFGCGKTVISQALSKYSNSDVIIYVGCGERGNEMSEVLRDFP      289
Drosophila_A2      VLDSLFPCVQGGTTAIPGAFGCGKTVISQALSKYSNSDVIIYVGCGERGNEMSEVLRDFP      289
                   ***:***.* *** .**************:********.::***********:*** :**

Saccharomyces      ELYTEM-SGTKEPIMKRTTLVANTSNMPVAAREASIYTGITLAEYFRDQGKNVSMIADSS      358
Cucumis            QLTMTLPDGREESVMKRTTLVANTSNMPVAAREASIYTGITLAEYFRDMGYNVSMMADST      354
Arabidopsis        QLTMTLPDGREESVMKRTTLVANTSNMPVAAREASIYTGITIAEYFRDMGYNVSMMADST      354
Homo               ELTMEV-DGKVESIMKRTALVANTSNMPVAAREASIYTGITLSEYFRDMGYHVSMMADST      351
Drosophila_A1      ELTCEI-DGVTESIMKRTALVANTSNMPVAAREASIYTGITLSEYFRDMGYNVAMMADST      348
Drosophila_A2      ELSVEI-DGVTESIMKRTALVANTSNMPVAAREASIYTGITLSEYFRDMGYNVSMMADST      348
                   :*   : .*  * :****:**********************::***** * :*:*:***:

Saccharomyces      SRWAEALREISGRLGEMPADQGFPAYLGAKLASFYERAGKAVALGSPDRTGSVSIVAAVS      418
Cucumis            SRWAEALREISGRLAEMPADSGYPAYLAARLASFYERAGKVKCLGGPERTGSVTIVGAVS      414
Arabidopsis        SRWAEALREISGRLAEMPADSGYPAYLAARLASFYERAGKVKCLGGPERNGSVTIVGAVS      414
Homo               SRWAEALREISGRLAEMPADSGYPAYLGARLASFYERAGRVKCLGNPEREGSVSIVGAVS      411
Drosophila_A1      SRWAEALREISGRLAEMPADSGYPAYLGARLATFYERAGRVKCLGNPEREGSVSIVGAVS      408
Drosophila_A2      SRWAEALREISGRLAEMPADSGYPAYLGARLASFYERAGRVKCLGNPEREGSVSIVGAVS      408
                   **************.*****.*:****.*:**:******:. .**.*:* ***:**.***

Saccharomyces      PAGGDFSDPVTTATLGITQVFWGLDKKLAQRKHFPSINTSVSYSKYTNVLNKFYDSNYPE      478
Cucumis            PPGGDFSDPVTSATLSIVQVFWGLDKKLAQRKHFPSVNWLISYSKYSSALESFYEKFDPD      474
Arabidopsis        PPGGDFSDPVTSATLSIVQVFWGLDKKLAQRKHFPSVNWLISYSKYSTALESFYEKFDPD      474
Homo               PPGGDFSDPVTSATLGIVQVFWGLDKKLAQRKHFPSVNWLISYSKYMRALDEYYDKHFTE      471
Drosophila_A1      PPGGDFSDPVTSATLGIVQVFWGLDKKLAQRKHFPSINWLISYSKYMRALDEYYDKNYPE      468
Drosophila_A2      PPGGDFSDPVTSATLGIVQVFWGLDKKLAQRKHFPSINWLISYSKYMRALDDFYDKNFPE      468
                   * *********:***.*.******************:*  :*****  .*:.:*:.   :

Saccharomyces      FPVLRDRMKEILSNAEELEQVVQLVGKSALSDSDKITLDVATLIKEDFLQQNGYSTYDAF      538
Cucumis            FISIRTKAREVLQREDDLNEIVQLVGKDALAEGDKITLETAKLLREDYLAQNAFTPYDKF      534
Arabidopsis        FINIRTKAREVLQREDDLNEIVQLVGKDALAEGDKITLETAKLLREDYLAQNAFTPYDKF      534
Homo               FVPLRTKAKEILQEEEDLAEIVQLVGKASLAETDKITLEVAKLIKDDFLQQNGYTPYDRF      531
Drosophila_A1      FVPLRTKVKEILQEEEDLSEIVQLVGKASLAETDKVTLEVAKLLKDDFLQQNSYSPYDRV      528
Drosophila_A2      FVPLRTKVKEILQEEEDLSEIVQLVGKASLAETDKITLEVAKLLKDDFLQQNSYSSYDRF      528
                   *  :* : :*:*.. ::* ::****** :*:: **:**:.*.*:::*:* **.:: ** .
```

```
Saccharomyces      CPIWKTFDMMRAFISYHDEAQKAVAN-------GANWSKLADSTGDVKHAVSSSKFFEPS      591
Cucumis            CPFYKSVWMMRNIIHFFNLANQAVERGAGMDGQKITYTLIKHRLGDLFYRLVSQKFEDPA      594
Arabidopsis        CPFYKSVWMMRNIIHFYNLANQAVERAAGMDGQKITYTLIKHRLGDLFYRLVSQKFEDPA      594
Homo               CPFYKTVGMLSNMIAFYDMARRAVETTAQS-DNKITWSIIREHMGDILYKLSSMKFKDPL      590
Drosophila_A1      CPFYKTVGMLRNIMAFYETARHAVESTAQS-DNKITWNTIRESMGGIMYQLSSMKFKDPV      587
Drosophila_A2      CPFYKTVGMLRNIIDFYDMARHSVESTAQS-ENKITWNVIREAMGNIMYQLSSMKFKDPV      587
                   **::*:. *:  :: :.: *.::*         .:. : .  *.: : : * ** :*

Saccharomyces      R-GEKEVHGEFEKLLSTMQERFAESTD---        617
Cucumis            E-GEPALIEKFKKLHEDLTNGFRALEDETR        623
Arabidopsis        E-GEDTLVEKFKKLYDDLNAGFRALEDETR        623
Homo               KDGEAKIKSDYAQLLEDMQNAFRSLED---        617
Drosophila_A1      KDGEQKIKADYDQLYEDLQQAFRNLED---        614
Drosophila_A2      KDGEAKIKADFEQLHEDLQQAFRNLED---        614
                   . **  :  .: :* . :   *    *
```

Figure S1. Comparison of amino acid sequences of V-ATPase subunit VHA-A from different species: *Saccharomyces cerevisiae*, *Cucumis sativus*, *Arabidopsis thaliana*, *Drosophila melanogaster* and *Homo sapiens*. Alignment was done in Clustal Omega. Cysteine residues are marked in yellow. The amino acid highlighted in turquoise is unique for *S. cerevisiae.*

***** (asterisk) indicates position of a single, fully conserved residue

: (colon) indicates conservation between groups of strongly similar properties (roughly equivalent to scoring > 0.5 in the Gonnet PAM 250 matrix)

. (period) indicates conservation between groups of weakly similar properties (roughly equivalent to scoring ≤ 0.5 and > 0 in the Gonnet PAM 250 matrix)

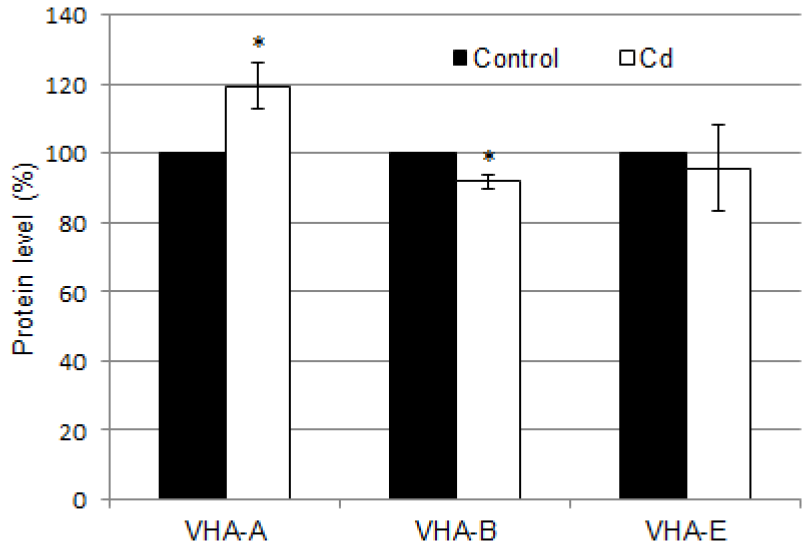

Figure S2. Average protein level of VHA-A, VHA-B and VHA-E V-ATPase subunits in roots of cucumber seedlings treated with 100 µM $CdCl_2$ for 24 h. The intensity of Western blot signals was analysed in ImageLab™. Results are presented as % of protein level measured under control conditions (100%). Data represent the means of 8 repetitions ± SE. Statistically significant differences (one-sample t test) between control and treatment are marked as * (p <0.05).
